# Cause-specific mortality in classic Kaposi's sarcoma: a population-based study in Italy (1995–2002)

**DOI:** 10.1038/sj.bjc.6605265

**Published:** 2009-08-25

**Authors:** V Ascoli, G Minelli, M Kanieff, R Crialesi, L Frova, S Conti

**Affiliations:** 1 Dipartimento di Medicina Sperimentale, Università La Sapienza, Viale Regina Elena 324, 00161 Rome, Italy; 2 Ufficio di Statistica, Istituto Superiore di Sanità, Viale Regina Elena 299, 00161 Rome, Italy; 3 Servizio Sanità e Assistenza, ISTAT, Viale Liegi 13, 00198 Rome, Italy

**Keywords:** CKS, multiple-causes-of-death, mortality, death certificate, epidemiology

## Abstract

**Background:**

Little information is available on the causes of death among persons with classic Kaposi's sarcoma (CKS).

**Methods:**

We conducted a population-based study in Italy to identify deceased persons with CKS and the underlying causes of death among them, by reviewing multiple-causes-of-death records. Standardised mortality ratios (SMRs) and 95% confidence intervals were calculated to compare the distribution of causes to that among the same-age general population of deceased persons. The geographical distribution was also evaluated.

**Results:**

Of the 946 deaths among persons with CKS, 65.9% were attributable to non-neoplastic conditions and 21.9% to malignancies. For 12.2%, no lethal pathology was identified and CKS was considered as the underlying cause. In 90% of these cases, there was visceral/nodal involvement, therapy-related complications, or neoplastic cachexia. Among persons with CKS who died of other causes, an excess for lymphoid malignancies emerged (SMR=4.40) (chronic lymphocytic leukaemia (11.03), non-Hodgkin's lymphoma (4.22), Hodgkin's lymphoma (11.80), and multiple myeloma (2.3)), balanced by a deficit for all solid cancers (0.56), with a marked deficit for lung cancer (0.41). We found an excess for respiratory diseases (chronic obstructive pulmonary disease (1.86)) and genitourinary diseases (chronic renal failure (6.47)). There was marked geographical heterogeneity in the distribution of deaths.

**Conclusions:**

Though referring specifically to Italy, the results are informative for other countries and populations and all cases of CKS in general.

Classic (or ‘Mediterranean’) Kaposi's sarcoma (CKS) occurs in elderly persons of mainly Mediterranean origin and is a rare outcome of infection with the human herpesvirus 8 (HHV8). Several studies have found an association (either positive or negative) between CKS and an additional primary cancer, both when CKS was the first primary neoplasm (Franceschi *et al*, 1996; Hjalgrim *et al*, 1997; Iscovich *et al*, 1999a) and when it was the second primary neoplasm (Iscovich *et al*, 1999b; Hisada *et al*, 2001), or regardless of the temporal order of diagnosis (Cannon *et al*, 2000). A few studies have also reported an association (positive or negative) with a variety of non-neoplastic diseases (Goedert *et al*, 2002; Guttman-Yassky *et al*, 2006; Anderson *et al*, 2008).

Classic Kaposi's sarcoma is rarely considered to be an actual cause of death (Brambilla *et al*, 1994; Franceschi *et al*, 1996; Hiatt *et al*, 2008). However, little information is available on the causes of death among persons with CKS. No population-based studies have been conducted, and the available information derives from the sparse data retrieved from case series (Brambilla *et al*, 1994; Lospalluti *et al*, 1995; Franceschi *et al*, 1996; Hiatt *et al*, 2008). Furthermore, whereas the 10th revision of the International Classification of Diseases (ICD-10) has a specific code for Kaposi's sarcoma (KS), the ICD-9, which in many countries is still used, does not.

The objectives of this study were to investigate cause-specific mortality among deceased persons with CKS and to determine whether the distribution of the underlying causes differs compared with the general population of deceased persons. To this end, we conducted a population-based study in Italy, which is one of the countries with the highest incidence rates of CKS in the Mediterranean basin and where HHV8 infection is endemic. Specifically, we reviewed multiple-causes-of-death records, which have never been used to study mortality among persons with CKS; we also evaluated the distribution by geographical area.

## Materials and methods

### Data sources

We used two data sources: the multiple-causes-of-death records and the National Mortality Database, both managed by Italy's National Census Bureau (ISTAT). The multiple-causes-of-death records are individual anonymous electronic records, which contain, in addition to the underlying cause (that is, the disease or injury that initiated the chain of events leading directly to death), all causes of death exactly as typewritten out in full by the medical examiner on the death certificate, in particular: (i) ‘other causes resulting in the underlying cause’, (ii) ‘other significant conditions’ (that is, those contributing to death yet not part of the chain of events leading directly to death), and (iii) the ‘immediate/final cause of death’ (that is, the final disease, injury or complication directly causing death) (Israel *et al*, 1986; Wall *et al*, 2005). The only geographical information collected in this database is the province of death. The National Mortality Database contains only the underlying cause of death, which until 2003 was coded using ICD-9 (KS codified as 173.9, ‘other malignant neoplasm of skin site unspecified’). It also contains the province of birth.

The analysis was conducted on data referring to the period from 1995 (that is, the year in which the multiple-causes-of-death records became available) to 2002 (that is, the year of the most recent available data). We excluded deaths in the provinces of Trento and Bolzano (both of which have small populations) because these provinces do not record the multiple causes written out in full.

### Identification of deceased persons with CKS

Using the multiple-causes-of-death records, we identified all persons for whom the term ‘Kaposi’ was written as any of the above-mentioned causes (*n*=1967, out of a total of 4 452 169 records). To exclude AIDS-related KS deaths, we carried out the following: (i) we eliminated the records with mention of HIV or AIDS anyplace on the death certificate; (ii) we manually reviewed the remaining individual records and excluded those with mention of AIDS-indicator opportunistic infections (WHO, 1994) (steps (i) and (ii) resulted in the elimination of 902 records); and (iii) we excluded all records for persons <65 years of age (*n*=89), limiting the analysis to older persons, who have an extremely low prevalence of HIV/AIDS (Dal Maso *et al*, 2005); in our records, only 4.5% of the decedents with KS and mention of HIV/AIDS were aged ⩾65 years. To exclude transplant-associated KS, we eliminated the records that mentioned transplants (*n*=30; 10 with heart transplant, 7 kidney, and 13 liver). The remaining 946 records were considered as ‘deaths with CKS’ and were used for this analysis.

### Causes of death among persons with CKS and differences compared with the general population

To identify the underlying cause of death of the 946 persons with CKS, we reviewed the individual records in the multiple-causes-of-death database. KS was recorded as the underlying cause on 486 (51.4%) death certificates. Of these, 275 (56.6%) had both KS and one or more lethal pathologies written as the underlying cause (for example, malignant tumours, circulatory system diseases, and respiratory system diseases). In these cases, the lethal pathology was considered as the underlying cause (as mentioned, CKS is rarely considered an actual cause of death); if more than one lethal pathology was written, we selected the most plausible cause based on an internationally accepted hierarchy of causes of death in ICD-9, which is used by ISTAT. In the remaining 211 (43.4%) of the 486 death certificates with KS as the underlying cause, KS was the only underlying cause; in these cases, we reviewed the ‘other causes resulting in the underlying cause’ to determine whether they included one or more lethal pathologies; this was the case in 95 of the 211 certificates (again, if more than one pathology was identified, the underlying cause was assigned based on the above-mentioned hierarchy). For the remaining 116 of the 211 certificates, no lethal pathology was found and KS was considered as the cause of death (death ‘due to’ or ‘caused by’ CKS). We then assigned the ICD-9 code to the 830 underlying causes of deaths, that is, excluding the 116 cases in which KS was the only identified cause (herein referred to as ‘KS alone’), for which no specific ICD-9 code exists.

To determine whether there were excesses or deficits for specific underlying causes of death among these 830 deceased persons with CKS, we compared the observed number of deaths with the expected number of deaths, calculated on the basis of the proportion of deaths due to that underlying cause among the same-age general population of persons who died in the same period (that is, given that the underlying cause is the only cause that is codified, it is the only cause that can be compared with the official statistics for the general population). The ratio of observed-to-expected cases can be considered as standardised mortality ratio (SMR)-like (herein referred to as ‘SMR’). The SMR was calculated for major groups of pathologies (for example, cardiovascular disease, respiratory disease) and for some specific pathologies (for example, specific cancers). The 95% confidence intervals (CIs) were calculated based on the Byar's formula, which provides accurate approximations when working with small numbers (Breslow and Day, 1987).

### Geographical distribution of deceased persons with CKS

For the geographical analysis, we calculated the SMR for the region of death, which was available for all 946 persons (Italy is divided into 20 regions, each of which is divided into provinces). Given that the area of birth is an important determinant because HHV8 is mostly acquired during childhood (Whitby *et al*, 2000; Cattani *et al*, 2003), we also calculated the SMRs for the region of birth (not recorded on the multiple-causes-of-death records), by linking the multiple-causes-of-death records with the National Mortality Database, using the individual anonymous six-digit identification code adopted for both databases. However, as the region of birth has been recorded in the National Mortality Database only since 1998, this linkage was only possible for 679 persons. Finally, for the regions of birth for which a significant SMR was found, we also calculated the SMRs by province of birth.

When calculating the SMRs for the region of death and region of birth, the reference population was Italy's entire general population for the same period. When calculating the provincial SMRs, the reference population was that of the single regions in the same period.

## Results

A total of 946 deaths among persons with CKS in the Italian population of persons ⩾65 years of age (range 65–101 years) were evaluated (619 in men and 327 in women). The distribution of age groups (that is, 65–74, 75–84, 85–94, and ⩾95 years) significantly differed by gender (Mann–Whitney test), with a median age of 82 years for men and 85 years for women. Only 171 (18.1%) persons were <75 years of age. When we carried out the analyses separately for men and women, no significant differences were found with respect to the results for the two genders combined.

Of the 946 deaths, 65.9% were attributable to non-neoplastic conditions, 21.9% to malignancies, and 12.2% to KS (that is, deaths for which no lethal pathology was identified). The SMRs and the 95% CI for the major groups of underlying causes of death are shown in Table 1. Compared with the general population, our study population had a marginal deficit for all cancers combined. There was also a deficit for diseases of the nervous system. When considering all cardiovascular diseases combined, a small deficit was found. This included a significant deficit for cerebrovascular disease (SMR: 0.52; 95% CI: 0.41–0.66) and ischaemic heart disease (SMR: 0.69; 95% CI: 0.56–0.85) (data not shown in the table), whereas there was a nearly two-fold excess for heart failure (SMR: 1.88; 95% CI: 1.26–2.68) and a small excess (though not significant) for hypertension (SMR: 1.22; 95% CI: 0.89–1.63).

There was an excess for both respiratory and genitourinary diseases. The excess for respiratory diseases was mainly attributable to chronic obstructive pulmonary disease (COPD) and allied conditions (SMR: 1.86; 95% CI: 1.45–2.37) and bronchopneumonia/pneumonia (SMR: 1.90; 95% CI: 1.28–2.74), whereas that for genitourinary diseases was mainly attributable to chronic renal failure (SMR: 6.47; 95% CI: 4.70–8.80) (data not shown in the table). There was no association for digestive diseases combined, whereas there was a non-significant deficit for cirrhosis (the most common digestive disease in our study) (SMR: 0.75; 95% CI: 0.39–1.31). Of the other causes, there was a deficit for senile dementia (SMR: 0.39; 95% CI: 0.18–0.74) and a slight deficit (though not significant) for diabetes (SMR: 0.68; 95% CI: 0.42–1.02).

For 116 (12.2%) death certificates, there was no mention of any lethal pathology; thus KS was considered as the cause of death (‘KS alone’). Of these cases, KS was disseminated or had visceral or nodal involvement (*n*=46; 39.7%), or there was mention of neoplastic cachexia (*n*=39; 33.6%) or senile decay or marasmus (*n*=10; 8.6%). In the remaining 21 (18.1%) cases, various complications were reported, including multiple skin ulcerations/necrosis (*n*=3), toxicity to chemotherapy or radiotherapy (*n*=2), lower-limb amputations (*n*=2), and anasarca (*n*=2), among others.

A primary cancer in addition to KS was recorded on 207 (21.9%) death certificates (144 men and 63 women) (Table 2). There was a deficit for all solid cancers combined. When considering specific sites, a deficit was found for lung, stomach and colon–rectum cancer; an excess, though not significant, was found for brain and prostate cancer.

For all malignant neoplasms of lymphatic and hematopoietic tissue, there was a more than four-fold excess, which was due to chronic lymphocytic leukaemia (CLL), Hodgkin's lymphoma (HL), non-Hodgkin's lymphoma (NHL), and multiple myeloma in both men and women.

Double primary cancers (that is, two cancers in addition to CKS) were recorded on 17 certificates (8.2%; 13 men and 4 women); 9 were solid-tumour pairs, 5 were solid/haematopoietic-tumour pairs (3 with CLL), and 3 were haematopoietic-tumour pairs (NHL/leukaemia; CLL/erythroleukaemia; CLL/Richter's syndrome).

Regarding the distribution by region of death, a wide heterogeneity emerged. There was an approximately three-fold excess for Sardinia, an approximately two-fold excess for Puglia and Calabria (southern Italy), a 1.5-fold excess for Lombardy (northern Italy), and a non-significant excess (1.6-fold) for Basilicata (southern Italy).

The distribution of deaths by region of birth (available for 679 persons) (Table 3) was similar to that by region of death, with a more than two-fold excess for Sardinia, Calabria, and Puglia, and a 1.3-fold excess for Lombardy. Basilicata (2.4-fold) and Sicily (1.3-fold) also showed a significant excess, and Campania (southern Italy) had a slight non-significant excess.

The analysis of SMRs by province of birth (data not shown in the table) revealed excesses for two provinces in Lombardy, in particular, Cremona (SMR: 4.33; 95% CI: 2.80–6.49) and Lodi (SMR: 5.98; 95% CI: 3.53–9.72); the Taranto province in Puglia (SMR: 1.69; 95% CI: 1.12–2.47); and the Matera province in Basilicata (SMR: 2.06; 95% CI: 1.03–3.71). In Sardinia, two provinces showed non-significant excesses, Sassari (SMR: 1.52; 95% CI: 0.91–2.37) and Oristano (SMR: 1.20; 95% CI: 0.44–2.61). A deficit was found for two provinces in Lombardy, that is, Varese (SMR: 0.42; 95% CI: 0.14–0.98) and Milano (SMR: 0.63; 95% CI: 0.43–0.88), and for the province of Catanzaro in Calabria (SMR: 0.28; 95% CI: 0.01–0.82).

## Discussion

The main findings of our study were that the leading underlying causes of death among deceased elderly persons with CKS were non-neoplastic conditions (65.9%), malignancies (21.9%), and ‘KS alone’ (12.2%) (that is, deaths for which CKS was assigned as the underlying cause). For non-neoplastic conditions, we observed an excess for respiratory and genitourinary diseases. Regarding malignancies, though there was an excess for all lymphoid malignancies combined, which is consistent with most previous studies (Iscovich *et al*, 1999a, 1999b; Cannon *et al*, 2000), it was balanced by a deficit for all solid cancers, also previously reported (Franceschi *et al*, 1996; Hjalgrim *et al*, 1997; Iscovich *et al*, 1999a, 1999b).

Regarding lymphoid malignancies, the 11-fold excess for CLL confirms the significantly increased risk of KS among persons with CLL (Iscovich *et al*, 1999b; Hisada *et al*, 2001). The four-fold excess for NHL is also consistent with previous studies (Iscovich *et al*, 1999a, 1999b; Cannon *et al*, 2000). Although no information was available on the site or type of NHL, one death for primary effusion lymphoma is of interest, given the rarity of this HHV8-associated neoplasm among HIV-negative persons (Ascoli *et al*, 2002). With respect to the nearly 12-fold excess for HL and the 2.2-fold excess for myeloma (borderline significance), again, our findings confirm previous reports (Iscovich *et al*, 1999b; Cannon *et al*, 2000; Serraino *et al*, 2007).

Regarding solid cancers, the deficit for lung cancer, also reported in other studies (Dictor and Attewell, 1988; Biggar *et al*, 1994; Hjalgrim *et al*, 1997; Iscovich *et al*, 1999a, 1999b; Goedert *et al*, 2002), could be explained by the inverse association between cigarette smoking and CKS (Anderson *et al*, 2008). However, our results are only in part consistent with this association, in which certain causes of smoking-attributable deaths showed a deficit (smoking-associated vascular mortality: ischaemic heart disease and cerebrovascular disease), whereas others showed an excess (smoking-related respiratory mortality: COPD and allied conditions). In any case, the literature on the association between CKS and cardiovascular and respiratory diseases is conflicting (Goedert *et al*, 2002; Guttman-Yassky *et al*, 2006; Anderson *et al*, 2008).

Of interest is the finding that 17 (8.2%) of the 207 persons with malignancies as the cause of death had double primary cancer. Although this number is small, ours is the first study to measure the occurrence of two independent primary cancers in separate organs in persons with CKS (previous studies were case reports) (Cottoni *et al*, 2002).

Although direct comparisons are difficult, the pattern of excesses/deficits for cancers in our study showed both similarities and differences when compared with persons with HIV/AIDS and transplant recipients (Grulich *et al*, 2007; Serraino *et al*, 2007; Dal Maso *et al*, 2009). In particular, we found a deficit for all cancers combined, compared with an excess reported for the other populations; lymphoid malignancies showed an excess in both our population and the others. Our deficit for lung cancer is in contrast to the excess for the other populations. The similarities for lymphoid malignancies could reflect immunological perturbations (immunodepression in persons with HIV/AIDS and transplant recipients and immunesenescence/inflammaging (Ostan *et al*, 2008) in elderly persons with CKS), whereas the differences for solid cancers could reflect different lifestyles/risk factors.

With regard to non-neoplastic diseases, the six-fold excess for chronic renal failure is consistent with the more than four-fold excess for kidney failure/dialysis in persons with CKS (Anderson *et al*, 2008), which, however, remains unexplained. Persons with chronic renal failure are at risk of developing HHV8-related diseases, and KS is a rare complication of glomerular diseases (Agbaht *et al*, 2007).

Concerning the considerable proportion of deaths (12.2%) that we assumed were actually caused by CKS, on >90% of these death certificates there were indications of the most aggressive forms of CKS (Brambilla *et al*, 2003), in particular, visceral/nodal involvement, complications related to therapy, or neoplastic cachexia (the remaining 10% were persons with senile decay). For the other persons with CKS yet who died of other causes, there was no indication of the specific stage of CKS, which is not recorded on death certificates. However, we can assume that CKS was not advanced, given that there was no mention of advanced disease anywhere on the death certificate. With regard to the length of survival, though this information is not available on death certificates, if we compare the median age at diagnosis in Italy (Dal Maso *et al*, 2005) to the median age at death in our study, we can roughly calculate that survival is ∼10 years, which is consistent with previous studies (Brambilla *et al*, 1994; Franceschi *et al*, 1996).

The geographical analysis showed a wide heterogeneity, though the reasons for the findings are unknown. In particular, our results confirmed that being born in southern Italy or the Islands is a risk factor for CKS (Geddes *et al*, 1995; Dal Maso *et al*, 2005), and we identified the specific regions in these areas. The results also revealed an excess for persons born in two adjacent provinces in the northern region of Lombardy, which is consistent with the increased incidence of CKS and high HHV8 seroprevalence in another adjacent province (Ascoli *et al*, 2001; Tanzi *et al*, 2005). These provinces are located in the Po Valley, which, together with Sardinia, Sicily, and Puglia, has shown evidence of a higher incidence of CKS and seroprevalence of HHV8, compared with other areas in Italy (Lospalluti *et al*, 1995; Calabro *et al*, 1998; Whitby *et al*, 2000; Cattani *et al*, 2003). This could be related to environmental risks, including having been born in areas with endemic malaria (Geddes *et al*, 1995) and exposure to bloodsucking insects (Coluzzi *et al*, 2002; Ascoli *et al*, 2006).

In drawing conclusions, some potential limits need to be mentioned. We excluded the 89 persons <65 years of age, some of whom may have been non-AIDS KS. Conversely, we may have included cases of iatrogenic (non-transplant) KS (that is, the 12 deaths for which the underlying cause was an autoimmune disease), though the relationship between CKS and iatrogenic KS is ambiguous and perhaps artificial. Regarding ascertainment bias, we are reasonably sure that there were no false positives, simply because of the peculiarity of the term ‘Kaposi’ and considering that in Italy KS has been diagnosed and reported as a cause of death for a number of decades using procedures that are homogeneous throughout the country. However, we cannot exclude the possibility of false negatives, a limitation that is inherent to death certificates (Redelings *et al*, 2007). Moreover, in comparing the two data sources, in the National Mortality Database some pathologies may be underestimated or overestimated, which would have an effect on our results. For certificates with both KS and another pathology as the underlying cause, we defined the lethal pathology as the cause, so that we may have underestimated the number of deaths for CKS alone and/or overestimated the number of deaths for other causes. Furthermore, some of the persons for whom the region of birth was not available may have been born outside Italy and acquired HHV8 infection abroad. However, this is extremely unlikely, given the advanced age of our population and that mass immigration in Italy is a very recent phenomenon. Finally, though not a limit of the study, it should be stressed that we used mortality data and that consequently not all persons with KS were identified. Nonetheless, it was not our objective to use mortality data as an indicator of prevalence.

To the best of our knowledge, this is the first population-based investigation of cause-specific mortality among deceased persons with CKS, which included a large number of cases (total of 946). Moreover, the results, which cover an extensive period, refer to Italy's entire population (about 57 million); previous population-based studies on CKS in Italy were based on incidence data provided by a network of cancer registries, which does not cover the entire country (Dal Maso *et al*, 2005). Though referring specifically to Italy, the results are also informative for other countries and populations and all cases of CKS in general.

## Figures and Tables

**Table 1:** Underlying causes of death among persons with classic Kaposi's sarcoma; Italy, 1995–2002

**Cause of death**	**ICD-9**	**Observed**	**Expected**	**SMR**	**95 CI%**
All cancers	140–239	207	241.7	0.86	0.74–0.98
Nervous system diseases^a^	320–359	8	22.0	0.36	0.16–0.72
Cardiovascular diseases^b^	390–459	343	419.6	0.82	0.73–0.91
Respiratory diseases^c^	460–519	128	67.6	1.89	1.58–2.25
Genitourinary diseases^d^	580–630	43	14.3	3.0	2.18–4.05
Digestive diseases^e^	520–579	29	41.1	0.71	0.47–1.01
Other causes^f^	—	72	—	—	—
Kaposi's sarcoma alone^g^	—	116	—	—	—
Total		946			

a Includes Parkinson's disease (3), encephalopathy (3), multiple sclerosis (1), and myelopathy (not otherwise specified) (1).

b Includes hypertensive disease (46), ischaemic heart disease (93), heart failure (30), cerebrovascular disease (68), other forms of heart disease (70), pulmonary embolism, (3) and other vascular diseases (33).

c Includes chronic obstructive pulmonary diseases/emphysema/asthma (68), bronchopneumonia (29), pneumothorax (2), pulmonary fibrosis (3), and other (26, of which 6 pulmonary oedema).

d Includes chronic renal failure (41) and urinary tract infection (2).

e Includes cirrhosis (12), ulcer (4), chronic hepatitis (5), intestinal occlusion (2), pancreatitis/biliary disease (3), ascites/peritonitis (2), and liver failure (1).

f Includes infectious and parasitic diseases (3), diabetes (22), autoimmune diseases (12) (for example, rheumatoid arthritis, polymyositis, systemic lupus erythematosus, and Sjögren syndrome), dementia (9), anaemia (16), bone marrow aplasia (5), and other (5).

g Death certificates without other underlying causes of death except Kaposi's sarcoma.

**Table 2:** Additional cancers among persons with classic Kaposi's sarcoma; Italy, 1995–2002

**Cancer type or site**	**ICD-9**	**Observed**	**Expected**	**SMR**	**95 CI%**
*All solid cancers*	140–199, 210–239	125	223.04	0.56	0.47–0.67
Stomach	151	6	18.79	0.32	0.12–0.69
Colon and rectum	153–154	15	26.40	0.57	0.32–0.94
Liver	155	7	8.20	0.85	0.34–1.76
Lung	161–162	19	46.50	0.41	0.25–0.64
Female breast	174	9	14.27	0.63	0.29–1.19
Prostate	185	19	13.32	1.43	0.86–2.23
Central nervous system^a^	191–192	7	3.10	2.29	0.90–4.65
Other sites^b^	—	25	—	—	—
Secondary neoplasms^c^	—	18	—	—	—
					
*Lymphatic and haematopoietic diseases*	200–208	82	18.65	4.40	3.50–5.50
Non-Hodgkin's lymphoma^d^	200, 202	28	6.63	4.22	2.81–6.13
Hodgkin's lymphoma	201	5	0.42	11.80	4.03–29.2
Multiple myeloma	203	9	3.97	2.27	1.03–4.27
Acute leukaemia^e^	204.0, 205.0	2	—	—	—
Chronic lymphocytic leukaemia^f^	204.1	22	1.99	11.03	6.90–16.70
Chronic myeloproliferative disorders^g^	—	4	—	—	—
Myelodysplastic syndromes	—	7	—	—	—
Lymphoproliferative disorders^h^	—	5	—	—	—
					
All cancers	140–239	207	241.7	0.86	0.74–0.98

a Includes glioma (2), glioblastoma (1), astrocytoma (3), and cerebral neoplasia (not otherwise specified) (1).

b Includes cancers of the oesophagus (2), pancreas (3), kidney (2), bladder (1), vulva/vagina (2), pleura (2), melanoma skin (1), non-melanoma skin (1), penis (2), gastrointestinal tract not otherwise specified (4), larynx (1), and uterus (4: 2 cervix and 2 endometrium).

c Includes liver, adrenal glands, lung, and brain metastasis of unknown primary sites.

d Includes cerebral lymphoma (1, age 75), primary effusion lymphoma (1, age 91), cutaneous lymphoma (1), and lymphoma (not otherwise specified) (2).

e Includes myeloid (1) and leukaemia (not otherwise specified) (1).

f Includes chronic lymphocytic leukaemia (21) and hairy cell leukaemia (1).

g Includes chronic myelogenous leukaemia (1), polycythemia vera (1), essential thrombocythemia (1), and chronic idiopathic myelofibrosis (1).

h Includes Waldenström's macroglobulinemia (1), Castleman's disease (1), gammopathy (not otherwise specified) (1), and lymphoproliferative diseases (not otherwise specified) (2).

**Table 3:** Mortality by region of birth among persons with classic Kaposi's sarcoma; Italy, 1998–2002

**Region**	**No. of deaths**	**Crude rates**	**Expected**	**SMR**	**95% CI**
*Northern*					
Piedmont	16	0.36	57.2	0.28	(0.16–0.45)
Valle d'Aosta	1	0.88	1.5	0.68	(0.01–3.7)
Liguria	12	0.60	26.1	0.46	(0.24–0.80)
Lombardy	132	1.65	103.9	1.27	(1.06–1.50)
Trentino-Alto Adige	3	0.38	10.2	0.29	(0.06–0.86)
Veneto	23	0.57	52.5	0.44	(0.28–0.66)
Friuli-Venezia Giulia	3	0.24	16.3	0.18	(0.04–1.35)
Emilia-Romagna	60	1.36	57.2	1.05	(0.80–1.35)
					
*Central*					
Marche	11	0.70	20.4	0.54	(0.27–0.96)
Tuscany	16	0.41	50.5	0.32	(0.18–0.51)
Umbria	4	0.43	12.1	0.33	(0.09–0.85)
Latium	14	0.31	58.8	0.24	(0.13–0.40)
					
*Southern*					
Campania	61	1.54	51.6	1.18	(0.90–1.52)
Abruzzi	13	1.02	16.5	0.79	(0.42–1.35)
Molise	3	0.89	4.4	0.68	(0.14–1.99)
Puglia	115	3.69	40.5	2.84	(2.34–3.41)
Basilicata	17	3.14	7.0	2.41	(1.4–3.89)
Calabria	58	3.44	21.9	2.65	(2.01–3.42)
					
*Insular*					
Sicily	72	1.74	53.7	1.34	(1.05–1.69)
Sardinia	45	3.53	16.6	2.72	(1.98–3.63)
Italy	679	1.30	679.0	1.00	
